# Discerning combining ability loci for divergent environments using chromosome segment substitution lines (CSSLs) in pearl millet

**DOI:** 10.1371/journal.pone.0218916

**Published:** 2019-08-28

**Authors:** Ramana Kumari Basava, Charles Thomas Hash, Mahesh D. Mahendrakar, Kavi Kishor P. B., C. Tara Satyavathi, Sushil Kumar, R. B. Singh, Rattan S. Yadav, Rajeev Gupta, Rakesh K. Srivastava

**Affiliations:** 1 International Crops Research Institute for the Semi-Arid Tropics (ICRISAT), Patancheru, Hyderabad, Telangana State, India; 2 Osmania University, Hyderabad, Telangana, India; 3 All India Coordinated Research Project on Pearl Millet (AICRP-PM), Indian Council of Agricultural Research (ICAR), Mandor, Jodhpur, Rajasthan, India; 4 Anand Agricultural University, Anand, Gujarat, India; 5 Institute of Biological, Environmental & Rural Sciences (IBERS), Aberystwyth University, Gogerddan, Wales, United Kingdom; Louisiana State University College of Agriculture, UNITED STATES

## Abstract

Pearl millet is an important crop for arid and semi-arid regions of the world. Genomic regions associated with combining ability for yield-related traits under irrigated and drought conditions are useful in heterosis breeding programs. Chromosome segment substitution lines (CSSLs) are excellent genetic resources for precise QTL mapping and identifying naturally occurring favorable alleles. In the present study, testcross hybrid populations of 85 CSSLs were evaluated for 15 grain and stover yield-related traits for summer and wet seasons under irrigated control (CN) and moisture stress (MS) conditions. General combining ability (GCA) and specific combining ability (SCA) effects of all these traits were estimated and significant marker loci linked to GCA and SCA of the traits were identified. Heritability of the traits ranged from 53–94% in CN and 63–94% in MS. A total of 40 significant GCA loci and 36 significant SCA loci were identified for 14 different traits. Five QTLs (flowering time, panicle number and panicle yield linked to Xpsmp716 on LG4, flowering time and grain number per panicle with Xpsmp2076 on LG4) simultaneously controlled both GCA and SCA, demonstrating their unique genetic basis and usefulness for hybrid breeding programs. This study for the first time demonstrated the potential of a set of CSSLs for trait mapping in pearl millet. The novel combining ability loci linked with GCA and SCA values of the traits identified in this study may be useful in pearl millet hybrid and population improvement programs using marker-assisted selection (MAS).

## Background

Pearl millet [*Pennisetum glaucum* (L.) R. Br.], commonly known as bulrush or cattail millet, is the most important small-seeded grain crop (i.e., millet). In its traditional growing areas, in India and many countries in sub-Saharan Africa, pearl millet grain is the staple food for poor households. In addition, pearl millet grain provides the staple diet for the urban poor, who value its high energy content and slow rate of digestion. Although pearl millet, is still largely used as a food crop in Africa and India, its grain is used for animal feed in Latin America (USA), and Australia. Pearl millet stems are used as a building material, fuel, and fodder for ruminant livestock. It is cultivated in areas with meager rainfall (300–500 mm), where other cereal crops such as maize or sorghum fail to grow. Therefore, pearl millet is an essential crop for the food security of the poor people in these dry areas.

The productivity of major crops is severely constrained by biotic and abiotic stresses. Drought limits crop production worldwide particularly in the semi-arid tropics and is projected to worsen with anticipated climate change. The significance of drought stress depends on its timing, duration, and intensity [1]. Drought stress during different growth stages is a common incidence in pearl millet affecting its yield [2]. Post-flowering drought stress reduced the pearl millet grain yield and yield stability [3]. Terminal drought stress (flowering through grain filling) is more harmful to pearl millet production than stress at vegetative crop growth stages. Asynchronous tillering and fast growth rate characters of this crop allow it to recover rapidly from uneven drought stress during the vegetative stage, but provide no benefits under constant terminal drought stress [3, 4]. Therefore, pearl millet breeding programs aimed at improving the adaptation of the crop to terminal drought stress environments in order to increase productivity and yield stability [5–10]. Most often, traits related to stress tolerance in crops are controlled by several genes with complex interactions among them as well as with the environment, making it difficult to unravel their genetic basis. Latest developments in crop physiology, efficient plant phenotyping and genomics provide a better understanding of the gene networks and novel tools to increase crop yield under drought [11]. QTL mapping is suitable to dissect complex phenotypic characters such as drought tolerance into their component traits, and to identify molecular markers linked to desired QTL alleles, which can be directly used in marker-assisted selection (MAS) [12–14]. Substantial research has been made in mapping QTLs for grain and stover-yield related traits under drought stress conditions in pearl millet [15, 16].

Hybrid cultivation, especially single-cross hybrids increased crop yields significantly. The introduction of hybrids increased grain yield from 305 kg ha^-1^ during 1951–1955 to 998 kg ha^-1^ during 2008–2012, that is about 300% productivity increase for pearl millet in India [17–18]. The knowledge of combining ability effects and the consequent variances is essential in selecting the parents and can be utilized in heterosis breeding to produce high yielding new recombinants. The yield performance of hybrids could not be calculated by the performance of their parents per se but by the combining ability of the parental lines [19]. So far, most studies of combining ability have aimed at the identification of promising parents [20, 21]. However, studies using genotypic data and combining ability performance would be essential to determine the real association between genetic distance and the heterosis effect and also to develop further strategies for breeding [20]. Fasahat et al. [22] reported that the capacity to estimate best genotype combinations for different traits based on molecular-based genetic data would significantly increase the effectiveness of plant breeding programs. The QTLs identified from the base population *per se* performance dataset may be different from those from GCA dataset [23]. The detection of better hybrid combinations depends on their parents combining ability and effects of genes that are involved in the expression of economically important quantitative and qualitative traits [24]. There are several studies of QTLs and gene analyses to identify the loci for combining ability and heterosis by using different molecular markers in several crops such as in rice, maize, sorghum, wheat, and rapeseed [25–33]. Line × tester analysis is one of the biometric procedures, commonly used to study combining the ability of the parents to be selected for heterosis breeding [34–36]. It also gives guidelines to find out the value of source populations and suitable recombination of traits in diverse genotypes to be used for the improvement of crop yield and its related traits. There have been several reviews and research on combining ability effects and their utilization on the selection of parents in breeding programs of pearl millet improvement [37–39]. Pearl millet whole genome was sequenced using a reference genotype Tift 23D2B1-P1-P5 and can be utilized to hasten pearl millet breeding and improving its genetic gains. Hybrid performance and marker-trait associations were established in 288 testcross hybrids of the pearl millet inbred germplasm association panel (PMiGAP) lines with ICMA 843–22 using the re-sequencing data [40]. However, in the case of pearl millet, there are no studies on discovering combining ability loci for grain and stover yield-related traits and unraveling its genetic basis.

Chromosome segment substitution lines (CSSLs) are a feasible alternative to recombinant inbred lines (RILs) and doubled haploid (DH) populations for precision mapping of QTLs and their interactions [41]. CSSLs are set of stable lines containing the entire information of the donor parent, while each CSSL carries one or more chromosome segments of donor parent in the genetic background of the recipient parent. Hence, it would have more advantages in the dissection of the genetic basis of complex traits. In pearl millet, a set of CSSLs was developed from advanced backcross populations of ICMB 841 and 863B [42]. The present study was aimed to estimate GCA and SCA effects of three testcross populations of pearl millet CSSLs for 15 grain and stover yield-related traits in irrigated (control) and moisture stress (MS) regimes through line × tester analysis and to identify QTLs associated with combining ability of these traits.

## Results

### ANOVA and correlation coefficients for combining ability values of grain and stover-related traits

ANOVA from line × tester analysis, mean, standard error (SE), coefficient of variation (CV%) and heritability (H^2^% on entry mean basis) for 15 grain and stover yield-related traits among three testcross hybrid populations of 85 pearl millet CSSLs and three testers in CN, MS and AMR are given in Table 1. The mean values of 255 testcrosses for all traits reduced in MS conditions when compared with CN. The percent coefficient of variations was more than 25% for GY, DSY, GNP, BM, PHI, GHI and VGI in both moisture regimes. However, in this experiment we had used extremely diverse testers that were crossed with CSSLs, resulting in a lot of genetic variability that posed challenges for accurate error variance estimates for some of the traits. Traits like biomass (BM), tiller number per plant (TN), dry stover yield (DSV) are extremely variable traits in pearl millet. These are influenced by many factors, especially plant densities and growth index. Heritability of traits in CN- ranged from 52–94% and in MS, it was 63–94%. All traits except TN showed high heritability (>70%) in both CN and MS.

**Table 1:** Analysis of variance, mean, CV (%), heritability (H^2^) from line × tester analysis of 85 CSSLs and three testers in control (CN), moisture stress (MS) and across two moisture regimes (AMR) in summer 2010.

Treatment/Source	DF	FT	PH	PL	PD	PN	TN	PY	GY	DSY	TGM	GNP	BM	PHI	GHI	VGI
**Control (CN)**																
Replication	2	10.273***	8.638***	1.8722	53.137***	3.208*	10.275***	6.547**	8.640***	6.785**	0.4218	1.4643	5.691**	4.078*	0.7508	4.685**
Hybrids	254	11.155***	3.674***	7.159***	4.704***	3.471***	1.675***	1.723***	4.392***	2.2221***	14.195***	2.557***	1.339***	3.157***	1.754***	1.581***
Lines	84	27.885***	6.893***	3.329***	4.766***	6.458***	1.467**	2.606***	4.073***	3.690***	5.831***	2.9034***	1.952***	2.334***	1.456***	2.823***
Testers	2	37.296***	44.367***	171.080***	102.128***	28.014***	4.301*	23.171***	167.450***	16.5632***	442.395***	72.952***	12.129***	109.957***	43.955***	9.405***
Line x tester	168	2.478***	1.581***	7.123***	3.513***	1.686***	1.748***	1.0263	2.611***	1.317***	13.280***	1.545***	0.904***	2.296***	1.401***	0.866***
Error	508															
Mean(SE)		42.94(0.83)	130.68(6.04)	20.29(0.72)	2.1(0.11)	308.12(34.77)	2.27(0.31)	3927.32(431.88)	2135.32(431.88)	2439.37(428.63)	6.96(0.32)	1016.36(173.22)	6082.05(1142.57)	0.52(0.07)	0.30(0.06)	115.70(21.90)
CV (%)		3.35	8.01	6.14	9.26	19.54	24.03	19.05	25.62	30.43	8.11	29.52	32.54	23.09	36.86	32.8
h^2^ (entry mean basis)		91	83	86	81	76	52	78	73	85	94	80	83	94	93	87
**Moisture stress (MS)**																
Replication	2	11.065***	9.560***	1.929	3.87*	2.6064	4.766**	3.2781*	8.235***	10.713***	9.873***	6.332**	4.237*	5.227**	7.727***	4.686**
Hybrids	254	16.597***	1.949***	5.781***	3.087***	5.485***	1.983***	2.360***	4.126***	3.862***	6.598***	3.336***	2.539***	4.254***	3.048***	3.137***
Lines	84	45.408***	2.954***	5.148***	2.329***	10.298***	1.468**	4.721***	3.186***	8.542***	6.225***	3.500***	5.377***	3.898***	3.305***	7.303***
Testers	2	36.084***	26.524***	88.001***	67.529***	53.437***	35.024***	22.913***	200.790***	6.674**	190.663***	121.480***	1.6533	174.294***	96.390***	0.7688
Line x tester	168	1.959***	1.154*	5.119***	2.699***	2.507***	1.847***	0.9358	2.255***	1.489***	4.594***	1.847***	1.130*	2.407***	1.808***	1.082
Error	508															
Mean(SE)		42.89(0.7)	133.08(6.07)	20.18(0.92)	2.08(0.15)	287.14(28.85)	2.02(0.24)	2468.91(299.24)	765.99(180.87)	1999.16(321.35)	4.7(0.37)	577.22(136.83)	4338.28(705.02)	0.3(0.06)	0.16(0.04)	83.06(13.51)
CV (%)		2.82	7.9	7.87	12.23	17.4	21.02	20.99	40.9	27.84	13.65	41.06	28.15	35.7	47.38	28.17
h^2^ (enrty mean basis)		94	70	90	85	85	63	77	75	87	86	86	85	92	93	88
**Across control and moisture stress regimes (AMR)**																
Treatment	1	0.06	2.55	0.33	0.02	18.54*	12.72*	829.44***	903.12***	43.19***	1034.55***	520.9***	675.63***	474.4***	349.04***	627***
Hybrids	254	25.84***	6.98***	15.76***	9.17***	9.55***	3.29***	15.99***	5.62***	13.11***	19.46***	9.13***	11.02***	23.3***	23.55***	14.72***
Treatment x Hybrids	254	0.96	1.86***	1.47***	2.29***	0.99	1.2*	4.38***	1.58***	1.62***	2.55***	2.35***	1.74***	5.1***	4.68***	1.57***
Lines	84	69.39***	13.28***	9.43***	5.35***	18.47***	3.12***	11.23***	10.46***	31.93***	10.36***	10.72***	26.09***	14.88***	21.7***	38.93***
Testers	2	164.71***	136.47***	1452.32***	815.49***	299.24***	162.69***	1256.17***	137***	184.48***	1895.99***	486.01***	138.82***	2019.93***	1836.1***	86.96***
Line x tester	168	2.42***	2.29***	1.83***	1.48***	1.65***	1.47***	3.6***	1.63***	1.67***	1.67***	2.66***	1.97***	3.74***	2.9***	1.75***
Treatment x line	84	0.85	3.12***	2.63***	4.79***	1.18	1.41*	4.74***	1.39***	1.8***	3.03***	2.63***	1.73***	8.29***	6.84***	1.51**
Treatment x tester	2	4.08*	6.79***	2.08	0.3	0.38	0.5	152.53***	16.32***	8.99***	95.4***	30.27***	16.67***	33.85***	27.64***	11.77***
Treatment x line x tester	168	0.98	1.16	0.88	1.07	0.9	1.11	2.44***	1.5**	1.45**	1.21*	1.87***	1.57***	3.16***	3.33***	1.47**
Error	1016															

**Note:** FT: Time to 75% flowering (d); PH: Plant height (cm); PL: Panicle length (cm); PD: Panicle diameter (cm); PN: Panicle number ('000/ha); TN: Tiller number per plant; TGW: 1000-Grain mass (g); GNP: Grain number/panicle; PY: Panicle yield (kg/ha); GY: Grain yield (kg/ha); DSY: Dry stover yield (kg/ha); BM: Biomass yield (kg/ha); PHI: Panicle harvest index (%); VGI: Vegetative growth index (kg/ha/d); GHI: Grain harvest index (%)

***Significant at 0.001 level of probability

** Significant at 0.01 level of probability

* Significant at 0.05 level of probability

There were significant differences for all sources of ANOVA for most of the traits in both CN and MS and also AMR with few exceptions. PY did not show a significant difference in the case of line × tester source in both moisture regimes and biomass did not show a significant difference between testers in MS regime. In MS, VGI did not show significant difference among testers and also in line x tester interactions. ANOVA of AMR showed significant differences in the interactions of treatment x line x tester in case of nine traits (PY, GY, DSY, TGM, GNP, BM, PHI, GHI, and VGI) and the remaining six traits (FT, PH, PL, PD, TN, and PN) did not show significant differences. There were significant differences in treatment × line interactions with respect to all traits except FT and PN and there were also significant differences in treatment x tester interactions with all traits except PL, PD, PN, and TN. The effect of treatments (CN and MS) was highly significant for nine traits i.e., PY, GY, DSY, TGM, GNP, BM, PHI, GHI, and VGI and significant for PN and TN.

General statistics of combining ability (GCA and SCA) for 15 grain and stover yield-related traits of testcross hybrids of 85 pearl millet CSSLs and three testers in CN, MS and AMR are given in Table 2. The GCA and SCA values of all the traits in CSSLs were normally distributed except for the GCA, SCA (H 77/833-2) and SCA (PPMI301) of plant height (PH) in CN conditions and SCA (H 77/833-2) and SCA (PPMI301) of PH and SCA (RIB 3135–18) of FT in MS conditions. The normal distribution of these GCA and SCA data sets have given the chance to identify genetic regions responsible for combining ability of traits through QTL mapping. The GCA and SCA values for many traits were more in CN treatment than that in MS condition. The correlation coefficients between the combining ability values of GY or DSY and those of other traits in CN, MS and AMR are given Table 3. Both GCA and SCA values of GY were positive and significantly correlated with those of five traits (PY, TGM, GNP, PHI, and GHI) whereas GCA and SCA values of DSY were positive and significantly correlated with BM in both CN and MS. However, GCA of GY was not significantly correlated with that of PHI in AMR. GCA and all three SCA values of GY correlated positively and significantly with the respective values of GNP under control and stress conditions, while across the environments, there was a significant negative correlation between GCA values of GY and GNP.

**Table 2:** Performance of GCA and SCA of 15 grain and stover yield-related traits in control (CN), moisture stress (MS) and across two moisture regimes (AMR) in summer 2010.

		GCA			SCA(H 77/833-2)			SCA(PPMI 301)			SCA(RIB 3135–18)		
Trait	Treatment	Minimum	Maximum	Skew	Minimum	Maximum	Skew	Minimum	Maximum	Skew	Minimum	Maximum	Skew
FT	Control	-2.83	5.17	0.59	-2.95	2.94	-0.55	-2.11	3.67	0.85	-2.94	1.84	-0.84
	Moisture stress	-2.66	5.34	0.70	-2.53	2.02	-0.39	-1.77	2.45	0.73	-2.26	1.08	-1.10
	AMR	-2.75	5.25	0.66	-1.29	1.87	0.68	-1.96	1.59	-0.70	-1.52	1.70	0.03
PH	Control	-29.46	15.32	-1.33	-33.93	21.73	-1.26	-30.52	19.48	-1.21	-11.32	16.68	0.74
	Moisture stress	-22.97	13.26	-0.76	-31.07	9.60	-2.96	-7.19	16.81	1.41	-7.96	14.93	1.01
	AMR	-14.81	10.92	-0.87	-8.10	8.07	0.24	-8.15	8.11	-0.13	-6.67	8.84	0.30
PL	Control	-2.18	1.71	-0.40	-1.39	1.06	-0.24	-3.38	3.07	-0.34	-2.46	3.54	0.43
	Moisture stress	-2.96	3.60	0.11	-1.53	2.92	0.76	-6.35	2.88	-0.69	-3.02	4.87	0.51
	AMR	-2.15	2.30	-0.30	-1.09	1.02	0.05	-1.67	1.22	-0.39	-1.07	1.32	0.41
PD	Control	-0.41	0.35	-0.40	-0.30	0.32	0.04	-0.40	0.41	-0.22	-0.30	0.29	0.04
	Moisture stress	-0.35	0.24	-0.09	-0.38	0.42	-0.02	-0.79	0.35	-0.74	-0.46	0.69	0.84
	AMR	-0.25	0.25	0.16	-0.16	0.19	0.56	-0.20	0.15	-0.70	-0.18	0.22	0.57
PN	Control	-116.23	96.21	-0.40	-65.73	61.83	-0.19	-71.95	102.83	0.46	-125.32	66.34	-0.70
	Moisture stress	-93.03	73.41	-0.36	-86.33	69.78	0.02	-73.00	99.23	0.34	-77.67	93.22	0.29
	AMR	-77.39	71.28	-0.35	-65.35	52.92	0.18	-41.96	43.31	-0.22	-43.90	60.04	0.02
TN	Control	-0.55	0.62	0.10	-0.72	0.80	0.00	-0.57	1.19	1.00	-1.35	0.77	-1.03
	Moisture stress	-0.34	0.45	0.03	-0.89	0.80	-0.05	-0.56	0.67	0.10	-0.86	0.57	-0.65
	AMR	-0.39	0.47	0.15	-0.38	0.53	0.58	-0.41	0.66	0.55	-0.40	0.34	-0.19
PY	Control	-997.43	762.02	-0.28	-1233.50	731.64	-0.78	-982.30	768.92	-0.53	-984.34	880.00	-0.16
	Moisture stress	-760.46	594.09	-0.26	-550.86	418.25	-0.32	-623.78	675.22	-0.21	-743.59	476.75	-0.20
	AMR	-449.80	697.60	0.41	-346.80	369.70	0.08	-389.50	605.10	0.19	-496.20	373.00	-0.35
GY	Control	-894.32	800.57	-0.01	-1004.60	725.72	-0.46	-650.85	614.26	-0.24	-870.76	1061.70	0.37
	Moisture stress	-470.22	403.23	-0.56	-367.66	388.67	-0.04	-486.93	574.29	0.01	-430.63	498.81	0.14
	AMR	-558.00	794.40	0.09	-468.80	396.50	-0.16	-382.80	523.40	0.18	-452.00	364.60	-0.58
DSY	Control	-886.26	1025.70	-0.14	-1041.20	1020.60	-0.15	-1158.90	1003.50	-0.33	-796.82	977.40	-0.23
	Moisture stress	-937.05	841.06	-0.28	-779.80	668.76	-0.40	-1071.40	809.40	-0.57	-720.94	599.95	0.01
	AMR	-802.30	734.30	-0.42	-356.20	494.00	0.49	-444.90	554.80	0.58	-410.20	391.30	-0.20
TGM	Control	-1.29	1.25	0.15	-1.51	1.35	-0.39	-1.71	1.54	-0.43	-1.89	2.70	0.54
	Moisture stress	-1.73	1.17	-0.79	-0.90	0.90	-0.15	-0.99	1.08	0.04	-1.40	1.55	0.33
	AMR	-1.45	1.07	-0.71	-0.43	0.58	0.50	-0.60	0.71	0.29	-0.53	0.81	0.38
GNP	Control	-271.69	511.75	0.78	-504.38	325.84	-0.78	-405.98	430.80	0.06	-367.31	529.25	0.68
	Moisture stress	-313.78	334.11	0.26	-287.21	454.57	0.24	-427.19	429.37	-0.18	-369.27	372.95	0.45
	AMR	-230.80	338.90	0.32	-181.74	250.53	0.36	-297.48	334.83	-0.02	-223.76	259.04	0.34
BM	Control	-1903.60	1879.30	-0.16	-2269.20	1796.40	-0.51	-2749.70	2081.60	-0.39	-1835.70	2425.20	-0.19
	Moisture stress	-1870.30	1545.10	-0.29	-1873.90	1114.10	-0.70	-1733.90	1454.40	-0.59	-1566.60	1047.20	-0.51
	AMR	-1396.20	1276.90	-0.29	-749.00	832.80	0.23	-751.20	854.60	0.41	-884.40	619.00	-0.53
PHI	Control	-0.18	0.12	-0.27	-0.21	0.15	-0.66	-0.17	0.14	-0.40	-0.20	0.21	0.43
	Moisture stress	-0.17	0.14	-0.40	-0.17	0.16	-0.39	-0.21	0.14	-0.67	-0.13	0.19	0.51
	AMR	-0.12	0.10	-0.39	-0.08	0.07	0.02	-0.08	0.09	-0.02	-0.08	0.08	-0.03
GHI	Control	-0.09	0.11	0.26	-0.15	0.11	-0.58	-0.12	0.10	-0.52	-0.17	0.17	0.41
	Moisture stress	-0.11	0.10	-0.20	-0.09	0.09	-0.36	-0.14	0.09	-0.73	-0.08	0.13	0.88
	AMR	-0.09	0.08	-0.23	-0.05	0.05	-0.02	-0.04	0.06	-0.01	-0.05	0.05	0.03
VGI	Control	-42.21	36.42	-0.19	-41.20	34.80	-0.40	-51.62	43.63	-0.40	-36.83	48.35	-0.18
	Moisture stress	-39.59	32.87	-0.32	-33.27	22.98	-0.55	-33.50	24.96	-0.67	-31.02	20.56	-0.53
	AMR	-32.50	27.42	-0.38	-18.43	17.41	-0.10	-14.17	16.49	0.45	-17.07	10.98	-0.67

**Note:** FT: Time to 75% flowering (d); PH: Plant height (cm); PL: Panicle length (cm); PD: Panicle diameter (cm); PN: Panicle number ('000/ha); TN: Tiller number per plant; TGM: 1000-Grain mass (g); GNP: Grain number/panicle; PY: Panicle yield (kg/ha); GY: Grain yield (kg/ha); DSY: Dry stover yield (kg/ha); BM: Biomass yield (kg/ha); PHI: Panicle harvest index (%); VGI: Vegetative growth index (kg/ha/d); GHI: Grain harvest index (%)

**Table 3:** Correlation analysis between GCA or SCA of grain yield or dry stover yield and 14 other yield-related traits in control (CN), moisture stress (MS) and across two moisture regimes (AMR) in summer 2010.

	GCA			SCA(H 77/833-2)			SCA(PPMI 301)			SCA(RIB 3135–18)		
Trait	Control	MS	AMR	Control	MS	AMR	Control	MS	AMR	Control	MS	AMR
**Grain yield**	** **	** **	** **	** **	** **	** **	** **					
FT	-0.539***	0.042	-0.753***	0.435***	0.311**	0.0983	-0.156	-0.125	0.236*	-0.389***	-0.436***	-0.1048
PH	0.064	0.14	0.378***	0.365***	0.360***	0.226*	0.15	-0.028	0.458***	0.072	0.273*	0.283**
PL	-0.259*	0.166	-0.400***	-0.006	-0.036	-0.0594	0.213	0.246*	-0.2019	0.796***	0.691***	0.007
PD	0.039	0.101	-0.0716	-0.455***	-0.194	-0.044	0.285**	0.423***	0.1467	0.597***	0.650***	0.121	
PN	0.45***	0.037	0.756***	0.470***	0.237*	0.423***	-0.05	-0.266*	0.0377	-0.518***	-0.517***	0.368***
TN	-0.371***	0.008	-0.487***	0.233**	0.089	0.357***	0.029	-0.246*	0.0546	-0.502***	-0.464***	0.270**
PY	0.812***	0.378***	0.737***	0.662***	0.548***	0.783***	0.800***	0.541***	0.798***	0.707***	0.553***	0.635***
DSY	0.464***	0.047	0.806***	0.502***	0.388***	0.537***	-0.056	-0.102	0.497***	0.003	-0.235*	0.392***
TGM	0.643***	0.695***	0.425***	0.745***	0.668***	0.179	0.319**	0.506***	0.260**	0.896***	0.839***	0.141
GNP	0.373***	0.638***	-0.361***	0.815***	0.822***	0.281**	0.705***	0.862***	0.496***	0.834***	0.921***	0.327**
BM	0.581***	0.198	0.924***	0.515***	0.398***	0.873***	0.229*	0.129	0.828***	0.323**	0.1	0.837***
PHI	0.864***	0.811***	0.0031	0.939***	0.941***	0.281*	0.850***	0.862***	0.544***	0.964***	0.950***	0.308**
GHI	0.560***	0.714***	-0.213*	0.794***	0.875***	0.337***	0.485***	0.772***	0.562***	0.854***	0.905***	0.367***
VGI	0.603***	0.168	0.908***	0.462***	0.355***	0.854***	0.255*	0.152	0.812***	0.367***	0.143	0.847***
**Dry stover yield**												
FT	-0.750***	-0.878***	-0.878***	0.357***	0.076	0.1989	0.489***	0.520***	0.250*	0.091	0.314**	0.1041
PH	0.464***	0.511***	0.541***	0.287**	0.206	0.301**	0.166	0.409***	0.426***	-0.072	-0.082	0.306**
PL	-0.232*	-0.567***	-0.532***	-0.011	0.059	-0.182	-0.558***	-0.490***	-0.099	-0.139	-0.273*	-0.040
PD	0.377***	-0.469***	-0.1865	-0.417***	-0.266*	0.0029	-0.592***	-0.510***	0.0227	-0.1	-0.230*	0.1248
PN	0.757***	0.880***	0.892***	0.152	0.285**	0.237*	0.436***	0.556***	0.0281	0.126	0.414***	0.1676
TN	-0.337**	-0.202	-0.569***	0.061	0.101	0.1215	0.299**	0.404***	0.0075	0.07	0.342**	0.1042
PY	0.652***	0.825***	0.361***	0.252***	0.371***	0.406***	0.03	0.171	0.506***	0.005	0.138	0.233*
GY	0.464***	0.047	0.806***	0.502***	0.388***	0.537***	-0.056	-0.102	0.497***	0.003	-0.235*	0.392***
TGM	0.239***	-0.244*	-0.0343	0.514***	0.308**	0.0725	-0.511***	-0.476***	0.1596	-0.044	-0.379***	0.1984
GNP	-0.353***	-0.574***	-0.697***	0.423***	0.265*	0.1799	0.005	-0.162	0.325**	-0.024	-0.256*	0.0763
BM	0.909***	0.972***	0.969***	0.851***	0.928***	0.859***	0.831***	0.883***	0.869***	0.828***	0.816***	0.800***
PHI	0.151	-0.428***	-0.413***	0.484***	0.345**	0.1154	-0.132	-0.062	0.383***	-0.046	-0.244*	0.1168
GHI	-0.03	-0.516***	-0.658***	0.661***	0.517***	-0.0949	0.266**	0.031	0.1869	0.311***	-0.146	-0.037
VGI	0.925***	0.975***	0.969***	0.829***	0.919***	0.809***	0.773***	0.859***	0.835***	0.803	0.803***	0.751***

**Note:** FT: Time to 75% flowering (d); PH: Plant height (cm); PL: Panicle length (cm); PD: Panicle diameter (cm); PN: Panicle number ('000/ha); TN: Tiller number per plant; TGW: 1000-Grain mass (g); GNP: Grain number/panicle; PY: Panicle yield (kg/ha); GY: Grain yield (kg/ha); DSY: Dry stover yield (kg/ha); BM: Biomass yield (kg/ha); PHI: Panicle harvest index (%); VGI: Vegetative growth index (kg/ha/d); GHI: Grain harvest index(%)

***Significant at 0.001 level of probability

** Significant at 0.01 level of probability

* Significant at 0.05 level of probability

### Identification of combining ability loci

A total 76 significant combining ability QTLs (with LOD> 5.0 and significance at p<0.001) were identified with both GCA and SCA values (all three testers) for15 grain and stover yield-related traits across CN, MS, and AMR. Out of 76 associations, only 5 were common in both GCA and SCA irrespective of moisture regimes whereas 11 were common in both CN and MS irrespective of GCA and SCA. Out of 88 markers, only 9 markers showed significant combining ability associations with traits and the GCA or SCA values of all traits (except TN) showed associations with at least one loci.

#### GCA loci

**Fig 1:**
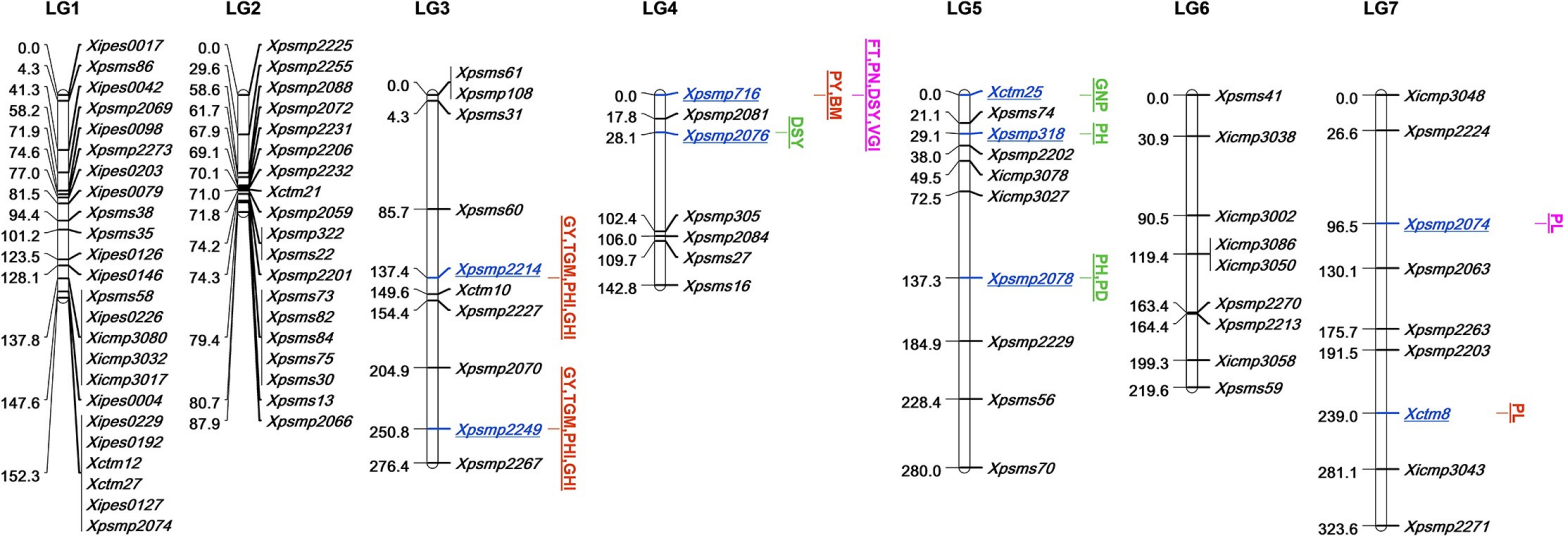
Linkage map of pearl millet showing significant GCA loci with LOD >5.0 and p<0.001. Significant GCA loci associated with traits in control (CN) (blue color), moisture stress (MS) (red color), CN+MS (pink color), CN+AMR (purple color), MS+AMR (green color) and CN+MS+AMR (maroon color) **Note:** FT-flowering time, PH- plant height, PL- Panicle length, PD- Panicle diameter, PN- Panicle number, PY- Panicle yield, GY- Grain yield, DSY- Dry stover yield, TGM- 1000-grain mass, GNP- grain number per panicle, BM- biomass yield, PHI- Panicle harvest index; GHI- grain harvest index, VGI-vegetative growth index.

A total of 40 significant GCA loci-trait associations were found with 14 traits in CN, MS, and AMR (Table 4 and their position on the linkage map is shown in Fig 1).

**Table 4:** Significant GCA loci with LOD value and additive effects for 15 grain and stover yield-related traits in control (CN), moisture stress (MS) and across two moisture regimes (AMR) in summer 2010.

GCA loci											
			Control			MS			AMR		
Trait	Chromosome	Marker	LOD	Additive effect	R^2^ value	LOD	Additive effect	R^2^ value	LOD	Additive effect	R^2^ value
FT	4	Xpsmp716	11.45	-2.191	46.22	13.75	-2.5	52.51	12.89	-2.35	50.26
		Xpsmp2076				8.95	-2.401	38.43			
PH	5	Xpsmp318	7.61	11.027	33.78						
		Xpsmp2078	8.24	10.989	36.01				8.20	7.34	35.85
PL	7	Xpsmp2074	5.15	2.215	20.86						
		Xctm8	6.31	0.582	28.98	5.26	0.853	24.79			
PD	5	Xpsmp2078	7.16	0.16	32.14						
PN	4	Xpsmp716	7.32	37.113	32.74	10.71	45.104	44.03	10.78	40.90	44.24
PY	4	Xpsmp716				8.08	284.2	35.45			
GY	3	Xpsmp2214				8.08	215.326	35.43			
		Xpsmp2249				8.08	215.326	35.43			
DSY	4	Xpsmp716	6.34	325.956	29.07	10.69	457.237	43.98	9.71	391.89	40.91
		Xpsmp2076	5.07	332.53	21.57						
TGM	3	Xpsmp2214				9.74	0.663	40.99	9.06	0.52	38.78
		Xpsmp2249				9.74	0.663	40.99	9.06	0.52	38.78
GNP	4	Xpsmp2076				5.24	-104.844	24.69			
	5	Xctm25	5.41	-196.582	25.42						
BM	4	Xpsmp716				10.34	0	42.9	9.29	605.55	39.54
PHI	3	Xpsmp2214				6.73	0.074	30.57	5.94	0.05	27.52
		Xpsmp2249				6.73	0.074	30.57	5.94	0.05	27.52
GHI	3	Xpsmp2214				5.48	0.045	25.69	6.02	0.04	27.81
		Xpsmp2249				5.48	0.045	25.69	6.02	0.04	27.81
VGI	4	Xpsmp716	6.36	14.595	29.16	10.98	17.944	44.84	10.50	15.54	43.37

**Note:** FT: Time to 75% flowering (d); PH: Plant height (cm); PL: Panicle length (cm); PD: Panicle diameter (cm); PN: Panicle number ('000/ha); TGM: 1000-Grain mass (g); GNP: Grain number/panicle; PY: Panicle yield (kg/ha); GY: Grain yield (kg/ha); DSY: Dry stover yield (kg/ha); BM: Biomass yield (kg/ha); PHI: Panicle harvest index (%); VGI: Vegetative growth index (kg/ha/d); GHI: Grain harvest index (%)

Out of which, five in CN, 5 in MS, one in both CN and MS, one in both CN and AMR, seven common in MS and AMR and four in all CN, MS and AMR were identified. Out of 15 traits, ten traits showed (FT, PH, PL, PY, GY, DSY, TGM, GNP, PHI, and GHI) had a maximum of two GCA loci each and TN had no GCA loci whereas the remaining four traits (PD, PN, BM, and VGI) showed at least one GCA locus. GY associated with two loci, *Xpsmp2214* and *Xpsmp2249* on LG3 and PY associated with one locus, *Xpsmp716* on LG4 particularly in only MS regime. Out of 88 markers, only 9 were associated with GCA values of traits. Marker *Xpsmp716* on LG4 showed the highest of 15 associations with six traits (PY in only MS regime, BM in MS and AMR and FT, PN, DSY, and VGI in all three in CN, MS, and AMR). This marker was followed by *Xpsmp2214* and *Xpsmp2249* on LG3 with seven associations each with four traits (GY, TGM, PHI, and GHI). The associations of three traits (TGM, PHI, and GHI) with these two markers were common in both MS and AMR whereas the remaining association of one trait, GY was observed only in MS. Out of 40 significant GCA loci-trait associations, 18 showed high additive effects (2.2–605.55), six showed non-additive effects (-2.19 to -196.58) and the remaining had very small additive or non-additive effects (<1.00). Out of five GCA loci observed only in MS, three loci (*Xpsmp2214* and *Xpsmp2249* on LG3 with GY and *Xpsmp719* on LG4 with PY) were due to high additive effects (285.2–215.32) and only one (*Xpsmp2076* on LG4 with GNP) was due to high non-additive effects (-104.844). The remaining one (*Xpsmp2076* on LG4 with FT) showed very small non-additive effects (-2.4).

#### SCA loci

**Fig 2:**
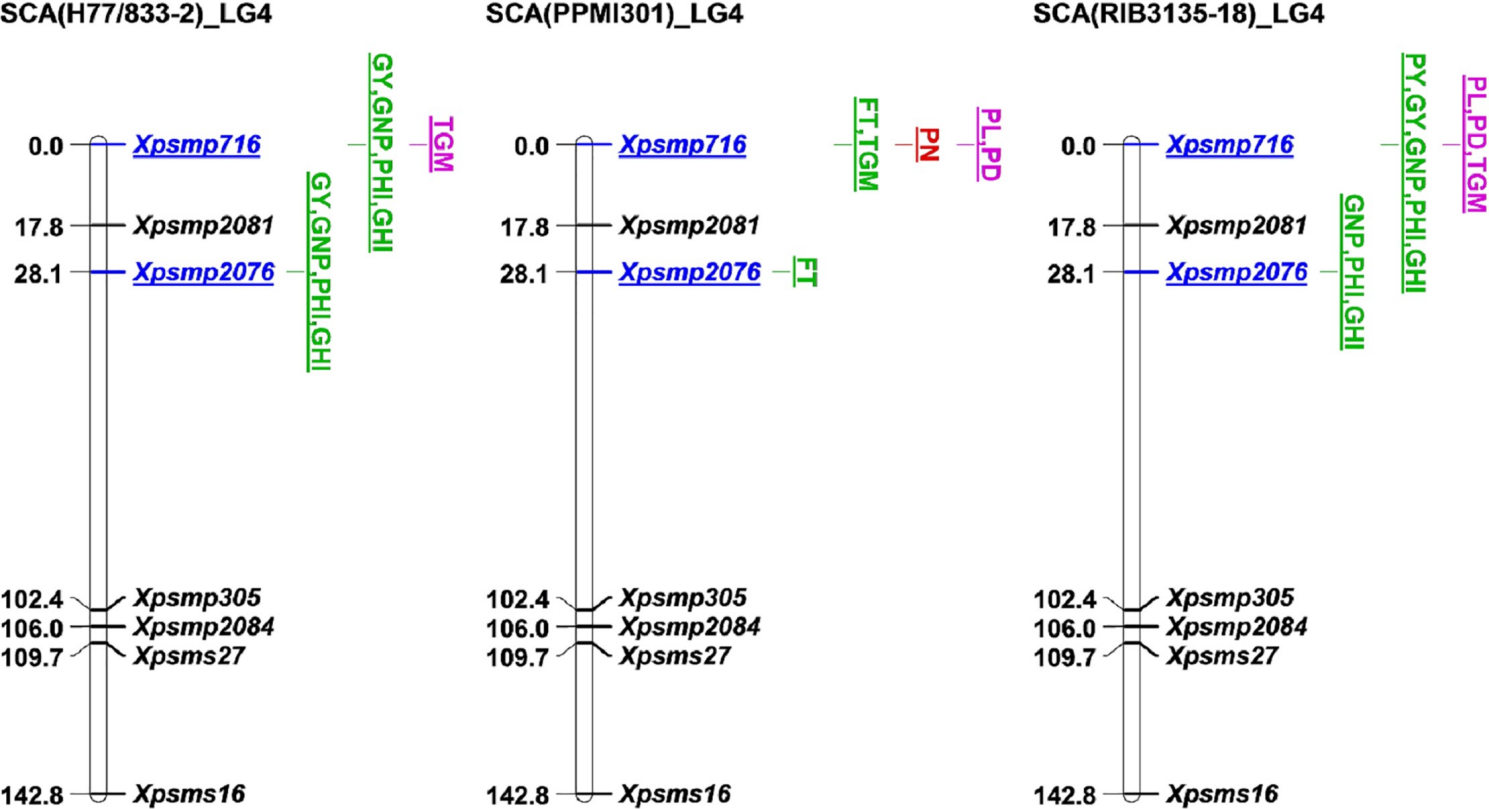
**Linkage map of pearl millet LG4 showing significant SCA loci with LOD > 5.0 and at p<0.001 with three testers a) H77/833-2; b) PPMI301;c) RIB3135-18.** Significant SCA loci associated with traits in control (CN) (blue color), moisture stress (MS) (red color), across moisture regimes (AMR) (green color), CN+MS (pink color), CN+AMR (purple color) and CN+MS+AMR (brown color) **Note:** FT-flowering time, PL- Panicle length, PD- Panicle diameter, PN- Panicle number, PY- Panicle yield, GY- Grain yield, TGM- 1000-grain mass, GNP- grain number per panicle, PHI- Panicle harvest index; GHI- grain harvest index.

Significant SCA loci-trait associations with their LOD values and additive effects in CN, MS and AMR are given in Table 5, and their position on the linkage map is shown in Fig 2. Total 36 SCA loci-trait associations with three testers were observed, out of which, ten associations with SCA (H 77/833-2), 12 with SCA (PPMI 301) and 14 were observed with SCA (RIB 3135–18). Only two markers, *Xpsmp716*, and *Xpsmp2076* on LG4 showed associations with 10 traits. There was only one association common with SCA of three testers i.e., *Xpsmp716* with TGM. This association was observed in both CN and MS regimes in the case of SCA (H 77/833-2) and SCA (RIB 3135–18) but it was observed only in CN in case of SCA (PPMI 301). However, in the case of AMR, marker-trait associations were observed with SCA values of only one tester, PPMI 301 and with remaining two testers there were no associations.

**Table 5:** Significant SCA loci with three testers, with LOD value and additive effects for 15 grain and stover yield-related traits in control (CN), moisture stress (MS) and across two moisture regimes (AMR) in summer 2010.

SCA loci											
			Control			MS			AMR		
Trait	Chromosome	Marker	LOD	Additive effect	R^2^ value	LOD	Additive effect	R^2^ value	LOD	Additive effect	R^2^ value
**SCA(H 77/833-2)**											
GY	4	Xpsmp716	9.91	355.69	41.55						
		Xpsmp2076	8.37	374	36.45						
TGM	4	Xpsmp716	7.76	0.38	34.34	7.47	0.34	33.28			
GNP	4	Xpsmp716	9.82	140.58	41.27						
		Xpsmp2076	9.26	154.26	39.43						
PHI	4	Xpsmp716	8.61	0.07	37.28						
		Xpsmp2076	6.56	0.07	29.93						
GHI	4	Xpsmp716	8.46	0.05	36.77						
		Xpsmp2076	7.88	0.05	34.76						
**SCA(PPMI 301)**											
FT	4	Xpsmp716	7.21	-0.91	32.34						
		Xpsmp2076	5.01	-0.88	23.79						
PH	4	Xpsmp716							5.283388	2.635	24.89
PL	4	Xpsmp716	7.79	1.42	34.42	10.59	1.69	43.66	3.176982	-0.322	15.81
PD	4	Xpsmp716	8.54	0.17	37.04	9.62	0.19	40.61			
PN	4	Xpsmp716				5.7	-27.63	26.59			
PY	4	Xpsmp716							3.391965	113.581	16.79
TGM	4	Xpsmp716	7.32	0.66	32.73						
PHI	4	Xpsmp716							3.181325	0.021	15.83
**SCA(RIB 3135–18)**											
PL	4	Xpsmp716	8.49	-1.44	36.87	10.05	-1.66	41.97			
PD	4	Xpsmp716	5.64	-0.11	26.33	7.05	-0.14	31.74			
PY	4	Xpsmp716	5.23	-235.06	24.68						
GY	4	Xpsmp716	10.15	-418.82	42.29						
TGM	4	Xpsmp716	8.99	-1.04	38.55	8.74	-0.68	37.72			
GNP	4	Xpsmp716	8.89	-154.4	38.21						
		Xpsmp2076	7.85	-165.04	34.64						
PHI	4	Xpsmp716	9.42	-0.08	39.97						
		Xpsmp2076	7.07	-0.08	31.81						
GHI	4	Xpsmp716	6.27	-0.05	28.79						
		Xpsmp2076	6.07	-0.06	28.04						

**Note:** FT: Time to 75% flowering (d); PH: Plant height (cm); PL: Panicle length (cm); PD: Panicle diameter (cm); PN: Panicle number ('000/ha); TGM: 1000-Grain mass (g); GNP: Grain number/panicle; PY: Panicle yield (kg/ha); GY: Grain yield (kg/ha); DSY: Dry stover yield (kg/ha); BM: Biomass yield (kg/ha); PHI: Panicle harvest index (%); VGI: Vegetative growth index (kg/ha/d); GHI: Grain harvest index (%)

Out of 10 significant associations detected in the SCA (H 77/833-2), only one association was common in both CN and MS regimes i.e., *Xpsmp716* with TGM. The remaining eight associations were observed only in control, with four traits (GY, GNP, PHI, and GHI), each linked to two loci *Xpsmp716* and *Xpsmp2076*. There were no associations particular to MS regime. The SCA effects of only two associations (*Xpsmp716* and *Xpsmp2076* with both GY and GNP) were due to high additive effects (140–374) and the remaining associations had negligible effects (0.05–0.38).

Out of 12 significant associations found with SCA (PPMI 301), one association was common in CN, MS, and AMR .i.e., *Xpsmp716* with PL. and one association (*Xpsmp716* with PD) was common in CN and MS. There were three associations in control (*Xpsmp716* with FT and TGM and *Xpsmp2076* with FT) and only one in MS (*Xpsmp716* with PN). Three associations were observed only in AMR (*Xpsmp716* with PH, PY, and PHI), Out of 12 associations, the SCA effect of only one association (*Xpsmp716* with PN in MS) was due to non-additive effect (-27.63) and another one (*Xpsmp716* with PY in AMR) was due to high additive effects (113.58). The remaining associations were due to either additive or non-additive but insignificant (-0.91 to +2.635).

Out of 14 significant associations detected with SCA (RIB 3135–18), there were three common associations in control and MS regimes, involving single marker, *Xpsmp716* with three traits PL, PD and TGM. The remaining eight associations were observed only in control, *Xpsmp719* with five traits, PY, GY, GNP, PHI and GHI and *Xpsms2076* with three traits GNP, PHI, and GHI. There were no associations particular to MS regime. In case of this tester, the SCA effects for all associations were due to non-additive effects, however, four associations (*Xpsmp716* with PY, GY and GNP and *Xpsmp2076* with GNP) have high non-additive effects ranging from -154.40 to -418.80.

### Performance of testcross hybrids of CSSLs and H 77/833-2

**Fig 3:**
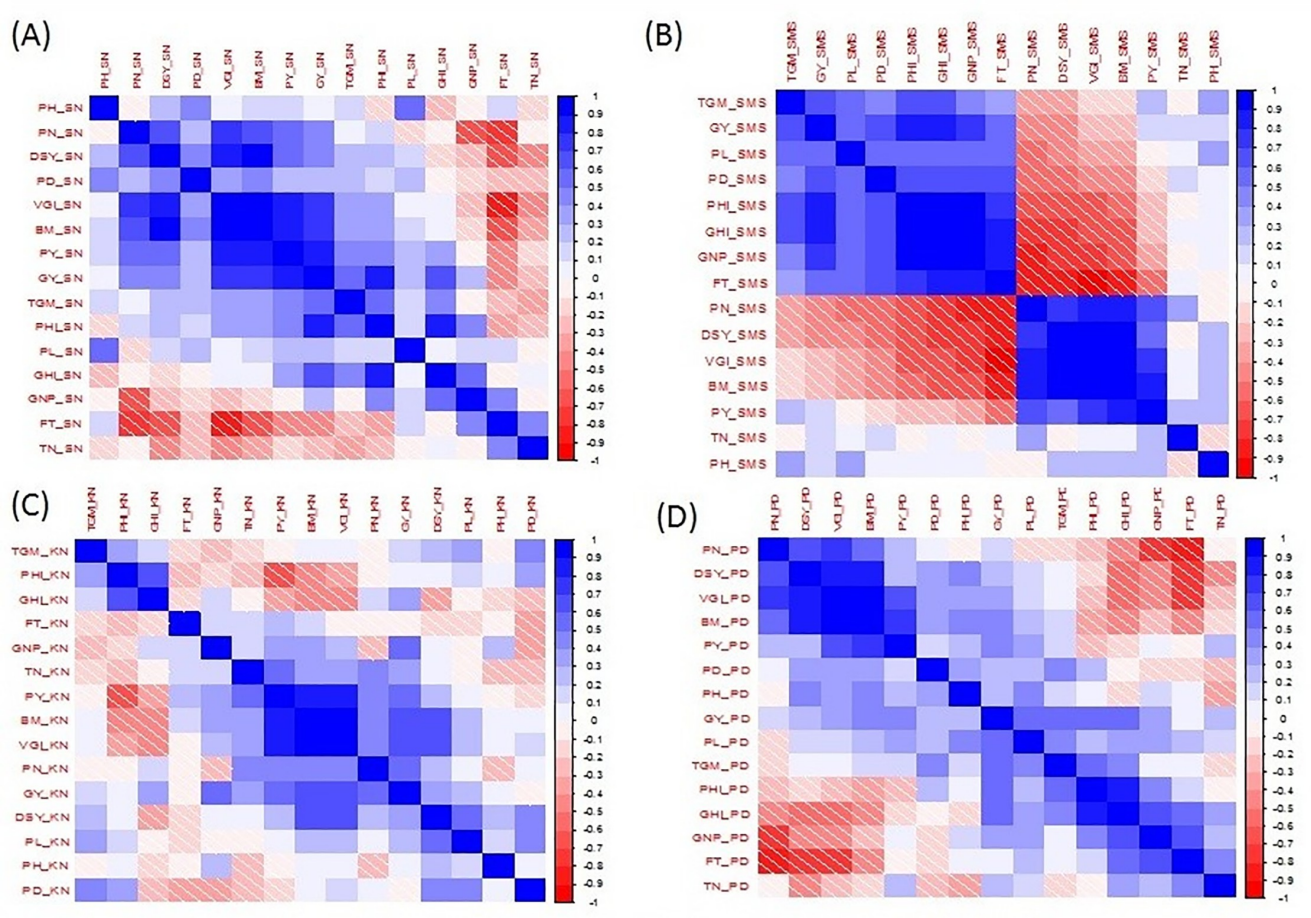
Heat map showing Pearson correlation plots among 15 traits in summer season control (SCN), summer season moisture stress (SMS), wet season control (WCN) in 2010 and for pooled data. **(A) Summer season control (SCN), (B) Summer season moisture stress (SMS), (C) Wet season control (WCN) in 2010 and (D) Pooled data. Note:** FT: Time to 75% flowering (d); PH: Plant height (cm); PL: Panicle length (cm); PD: Panicle diameter (cm); PN: Panicle number ('000/ha); TN: Tiller number per plant; TGW: 1000-Grain mass (g); GNP: Grain number/panicle; PY: Panicle yield (kg/ha); GY: Grain yield (kg/ha); DSY: Dry stover yield (kg/ha); BM: Biomass yield (kg/ha); PHI: Panicle harvest index (%); VGI: Vegetative growth index (kg/ha/d); GHI: Grain harvest index (%); Bar on right side of plot represents correlation value.

Evaluation testcross hybrids of 85 CSSLs and H 77/833-2 were also performed in wet season 2010 only under fully irrigated conditions. Descriptive statistics for 15 grain and stover yield traits under summer season control (SCN), summer season moisture stress (SMS), wet season control (WCN) in 2010 and for pooled data of these three treatments are shown in Table 6. The minimum, maximum and mean values for all traits were higher in WCN than these parameters for all traits in the other two treatments (SCN and SMS). The mean value of FT was more by one in WCN indicating late flowering by one day as expected during the rainy season. Almost all traits followed a normal distribution in all treatments. Pearson correlation among 15 traits computed separately in three treatments (SCN, SMS, and WCN) and for pooled data (PD) (Fig 3). In SCN and WCN, BM was positively and significantly correlated with GY whereas, in SMS, BM was negatively and significantly correlated with GY. All traits were positively correlated with GY in WCN, except PD. Dry stover yield was positively and significantly correlated with BM and PY but negatively and significantly with GHI in all three treatments and pooled data analysis.

**Table 6:** Descriptive statistics of testcross hybrids of 85 CSSLs and H 77/833-2 under summer season control (SCN), summer season moisture stress (SMS), wet season control (WCN) in 2010 and for pooled data.

Source	FT	PH	PL	PD	PN	TN	PY	GY	DSY	TGM	GNP	BM	PHI	GHI	VGI
**Summer season control (SCN)**															
Mean	43.93	135.89	18.95	1.90	337.69	2.46	4160.70	2534.50	2888.90	6.62	1172.60	7037.90	0.61	0.36	131.47
Minimum	40.00	113.33	16.33	1.60	214.33	1.60	3443.30	1806.70	1979.30	5.23	805.33	5446.00	0.51	0.28	91.77
Maximum	50.67	153.33	20.33	2.44	472.33	3.63	5165.00	3377.70	4227.70	7.82	1952.30	9440.50	0.71	0.45	184.31
SD	3.23	8.81	0.75	0.15	55.64	0.36	401.79	393.54	462.22	0.46	199.66	789.69	0.05	0.04	19.93
C.V.	7.34	6.48	3.97	7.61	16.48	14.80	9.66	15.53	16.00	6.92	17.03	11.22	8.76	10.40	15.16
Skew	0.63	-0.55	-0.67	0.84	-0.13	0.48	0.46	0.25	0.19	0.24	1.03	0.44	0.17	0.18	0.04
Kurtosis	-1.05	-0.13	1.17	2.46	-0.86	0.49	-0.26	-0.85	-0.04	0.46	1.92	0.25	-1.00	-0.23	-0.61
**Summer season moisture stress (SMS)**															
Mean	43.60	136.47	18.98	1.89	318.01	2.20	2541.50	887.57	2307.20	4.68	634.35	4858.60	0.35	0.19	91.61
Minimum	40.00	121.00	16.33	1.66	203.33	1.53	1814.70	225.00	1362.70	3.16	197.00	3258.00	0.12	0.06	55.26
Maximum	49.00	153.67	22.67	2.19	446.00	3.25	3257.30	1353.30	3221.30	5.96	1100.00	6167.70	0.56	0.35	120.93
SD	3.01	5.92	1.12	0.13	62.81	0.28	324.51	250.38	508.67	0.62	226.24	743.29	0.11	0.07	18.01
C.V.	6.91	4.34	5.89	6.62	19.75	12.69	12.77	28.21	22.05	13.17	35.67	15.30	30.33	37.10	19.66
Skew	0.57	0.30	0.06	0.25	-0.14	0.57	-0.19	-0.22	-0.30	-0.61	0.44	-0.23	0.17	0.40	-0.27
Kurtosis	-1.34	0.14	0.39	-0.70	-1.04	1.82	-0.81	-0.42	-1.22	-0.13	-0.90	-1.09	-0.99	-1.00	-1.31
**Wet season control (WCN)**															
Mean	44.51	174.87	21.01	1.97	257.28	2.82	4401.60	2966.10	3984.90	6.95	1675.90	8341.80	0.68	0.36	153.30
Minimum	41.33	162.67	19.33	1.65	185.00	2.00	2756.00	1814.00	2741.30	5.36	1246.00	6519.00	0.35	0.22	117.55
Maximum	47.33	195.00	24.00	2.34	335.33	3.94	7731.30	4641.50	6853.30	8.41	2389.00	13196.00	0.76	0.43	231.50
SD	1.32	6.47	0.82	0.15	30.67	0.38	1023.90	474.24	663.89	0.54	229.11	1387.70	0.09	0.04	25.30
C.V.	2.97	3.70	3.89	7.40	11.92	13.36	23.26	15.99	16.66	7.79	13.67	16.64	13.00	10.38	16.51
Skew	-0.05	0.69	0.68	0.18	0.03	0.72	1.57	0.46	1.22	0.00	0.75	1.07	-2.28	-1.03	0.91
Kurtosis	-0.60	0.68	1.62	-0.06	-0.07	0.87	2.23	0.74	3.05	0.71	0.56	1.00	4.43	1.87	0.51
**Pooled data**															
Mean	44.01	149.08	19.64	1.92	304.33	2.49	3701.30	2129.40	3060.30	6.08	1161.00	6746.10	0.55	0.30	125.46
Minimum	40.00	113.33	16.33	1.60	185.00	1.53	1814.70	225.00	1362.70	3.16	197.00	3258.00	0.12	0.06	55.26
Maximum	50.67	195.00	24.00	2.44	472.33	3.94	7731.30	4641.50	6853.30	8.41	2389.00	13196.00	0.76	0.45	231.50
SD	2.68	19.62	1.32	0.14	61.77	0.42	1058.20	975.48	887.53	1.14	478.59	1760.30	0.17	0.09	33.25
C.V.	6.08	13.16	6.74	7.44	20.30	17.02	28.59	45.81	29.00	18.77	41.22	26.09	30.23	31.34	26.50
Skew	0.44	0.51	0.16	0.48	0.31	0.60	0.73	-0.25	0.58	-0.58	0.04	0.42	-0.67	-0.86	0.22
Kurtosis	-0.86	-1.10	-0.03	0.64	-0.91	0.54	1.45	-1.14	0.79	-0.55	-0.79	0.34	-0.76	-0.49	0.00

**Note:** FT: Time to 75% flowering (d); PH: Plant height (cm); PL: Panicle length (cm); PD: Panicle diameter (cm); PN: Panicle number ('000/ha); TGM: 1000-Grain mass (g); GNP: Grain number/panicle; PY: Panicle yield (kg/ha); GY: Grain yield (kg/ha); DSY: Dry stover yield (kg/ha); BM: Biomass yield (kg/ha); PHI: Panicle harvest index (%); VGI: Vegetative growth index (kg/ha/d); GHI: Grain harvest index (%)

## Discussion

Pearl millet is an important climate-resilient crop for the dry and marginal regions that can grow in low input conditions [43, 44]. In addition to the open-pollinated varieties (OPVs), pearl millet has well developed CMS-based hybrid systems and has a substantial area under single-cross hybrids. Development of high yielding seed and restorer parents with good *per se* performance and generation of information on GCA and SCA has been an important exercise in pearl millet hybrid breeding programs globally [45].

Generally, selection of plants from the population is done by evaluating the average performance of their testcrosses, and the plants showing hybrid vigor in the testcrosses are then inter-mated to form the next population in breeding programs [29]. Establishing heterotic groups based on combining ability patterns appears to be the most effective and efficient approach for pearl millet improvement. A two-step selection procedure based on both GCA and SCA might be preferable to estimate hybrid performance rather only based on GCA [20]. The present study also concluded that the genetic basis of GCA and SCA varies based on their marker-trait association and hence both GCA and SCA are equally important for pearl millet heterosis breeding. Several favorable alleles associated with combining ability were pyramided by marker-assisted selection, and the combining ability of the selected lines was developed in rice [26].

In the present study, 255 testcross hybrids, produced by using a set of 85 CSSLs and three elite pollinators were evaluated for 15 grain and stover yield-related traits in control (CN) and moisture stress (MS) regimes. Line × tester analysis was performed to estimate their GCA and SCA values in CN, MS and also across two moisture regimes (AMR). Combining ability studies have been conducted for agronomic traits not only under normal environments but also for abiotic and biotic stress environments, such as in maize for drought stress tolerance [28, 46, 47] and in cassava for anthracnose disease resistance [48]. In most cases, GCA and SCA of all 15 traits in the CSSLs exhibited a normal distribution in all three instances (CN, MS, and AMR). Hence, genetic loci associated with GCA and SCA of traits could be identified using QTL mapping. As most of the QTL mapping methods make use of the assumption that the quantitative phenotype follows a normal distribution. The normal distribution of GCA and SCA variance components indicate the occurrence of numerous small-effect genetic loci and their interactions with the environmental factors. Since the GCA and SCA variance components had random variables with a Gaussian distribution, the frequencies of lines with higher GCA and SCA values for the mentioned traits were reactively less. The increase or decrease in the mean GCA and SCA values for traits was not consistent with the treatment. However, the mean values of GCA for GY and DSY were more in MS treatment. Mhike et al. [46] also reported that GCA for grain yield and ears per plant in maize under drought were higher than those of in well-watered environments.

Based on GCA effects of all 15 traits (S1 Table), the top ten genotypes with high positive significant GCA effects for GY (> 538) in control conditions also possessed high positive and significant GCA effects for PY and TGM, while in MS conditions, the top nine genotypes with high positive significant GCA effects for GY (> 220) also had high positive and significant GCA effects for DSY and BM. In the case of AMR, the top ten genotypes with high positive GCA effects for GY (>380) also showed high positive GCA effects for PY, DSY, TGM, BM, and VGI. These results indicate that in control, most of the carbohydrate reserves were mobilized towards more to grain formation than in MS, where grain formation was reduced due to moisture stress and more biomass was accumulated. The variations in GCA and SCA values of traits suggest the complex genetic basis for GCA and SCA. Correlation analysis between GCA and SCA of GY or DSY with that of other traits revealed that the combining ability values of GY were strongly correlated with those of PY, TGM, GNP, PHI, and GHI while combining ability of DSY with that of BM. These results indicate that both grain number and mass along with panicle yield could be considered over other traits such as FT, PH, PN, and PH, etc., for genetic enhancement of GY and similarly, for DSY, BM (indirectly FT) could be given more attention. Yadav et al. [5] reported that significant positive association of grain yield with grain mass, grain number, panicle number, biomass, panicle harvest index and grain harvest index whereas stover yield showed a negative correlation with grain mass in pearl millet. Panicle harvest index, grain number, and mass were highly correlated with grain yield in pearl millet [2]. Early flowering plants usually escape from terminal drought by decreasing growth duration [49]. Production of a large number of tillers provides potential compensation for damage to the main shoot or primary tillers during mid-season drought stress but can increase susceptibility to terminal moisture stress [3–4].

The average GY of testcross hybrids (85 CSSLs x H 77/833-2) recorded in summer control (SCN), wet season control (WCN) was 2534 kg/ha and 2966 kg/ha respectively. The average GY of pooled data of three treatments (SCN, SMS, and WCN) was 2129.4 kg/ha. These results indicated the stable performance of CSSLs across two seasons. Correlation results among GCA values of traits are compared with correlation results among trait values of testcross hybrids of CSSLs and H 77/833-2 in three treatments (SCN, SMS, and WCN) and also their pooled data. In summer control conditions, GY correlated positively and significantly with almost all traits except FT and TN in both correlations. However, in summer moisture stress conditions, there were few differences like correlation using GCA values of traits showed a positive correlation of GY with all traits whereas CSSLs and H 77/833-2 testcrosses results had a negative correlation of GY with PN, DSY, and BM. Similarly, in the case of DSY, these correlations results were in harmony with both control and moisture stress conditions in summer. For example, DSY was correlated positively and significantly with BM and PY in correlation results using GCA values as well as with absolutes values of testcross hybrid.

In this maiden attempt to understand the genetic basis of combining ability, it was anticipated that GCA and SCA were mainly connected to the additive and non-additive genetic effects, respectively [50]. Combining ability can be transmitted and accrued over generations [51]. These reports supported the use of molecular markers to unravel the genetic basis of GCA or SCA similar to the traits of yield and yield components per se. Loci linked to GCA have been identified using different mapping populations including DHs, RILs, BCRILs, and ILs in different crops [23, 26, 29]. QTLs linked to GCA values of 10 agronomic traits have been detected in three testcross populations developed from three testers and recombinant inbred lines (RIL) and backcross recombinant inbred lines (BCRIL) in rice [30]. Qi et al. [29] reported several GCA and SCA loci for yield-related traits using a set of testcross hybrids of introgression lines (ILs) of maize under different environmental conditions. Liu et al. [26] reported two major combining ability genes, *OsPRR37*, and *Ghd7* for flowering time, plant height and spikelets per plant in rice using BC_3_F_2_ population and a set of near-isogenic lines.

In the present study, 40 significant associations with GCA values and 36 with SCA values of 15 grain and stover yield-related traits were identified altogether in CN, MS, and AMR. Only 5 associations (8.33%) (FT, PN, and PY with *Xpsmp716*, FT, and GNP with *Xpsmp2076* on LG4) were common to both GCA and SCA suggesting that the genetic basis of GCA and SCA were different. This is in agreement with statements of earlier reports of Sprague and Tatum, 1942 [52] on associations to both control and MS. These results indicated that the genetic basis of combining ability loci is also different in two moisture regimes. Huang et al. [28] identified 16 loci for the GCA of yield per plant in maize introgression lines, out of which, only bnlg1017 was common in two environments. He also reported that allele from donor parent increased the GCA of yield per plant across various environments. The present study showed that the presence of alleles from donor parent 863B at significant GCA loci increased the GCA values of the respective traits. These results agree with the earlier reports [50–52] that GCA was caused by additive effects and SCA by non-additive effects. Belicuas et al. [27] published four QTLs with additive effects for stay-green trait in maize and emphasized the importance of additive effects than dominant effects in heterosis.

Significant GCA loci for GY were not observed in CN and AMR but GCA value of GY in MS regime was significantly associated with two loci, *Xpsmp2214*, and *Xpsmp2249* on LG3. The GCA values at these two loci were due to high additive effects and the contributing alleles are from donor parent 863B. Hence, these two loci are highly important in pearl millet heterosis breeding for improving grain yield under drought conditions. In the case of SCA for GY, there were two significant loci (*Xpsmp716* and *Xpsmp2076* on LG4) associated with its SCA (H 77/833-2) with additive effects and one locus, *Xpsmp716* with non-additive effects with SCA (RIB 3135–18) only in control. There were no SCA loci for GY in MS regime. Only one GCA locus, *Xpsmp716* was significantly associated with GCA values for DSY in all three instances, CN, MS, and AMR. This marker could be useful further in breeding programmes of pearl millet where it is grown especially for fodder to livestock. There were no significant SCA loci observed with this trait.

There were two GCA loci (*Xpsmp2214* and *Xpsmp2249)* linked to three traits each (TGM, PHI, and GHI) common in both MS and AMR, which can be considered as stable drought tolerance loci for improving these three respective traits. Marker, *Xpsmp716* can be considered as stable GCA locus for four traits (FT, PN, DSY, and VGI) as this marker was linked to GCA values of these traits in all three occurrences of CN, MS, and AMR. Out of 88 markers, only nine were linked with GCA and SCA values of 14 traits (out of 15 traits). The simultaneous effects of each combining ability loci on multiple traits in all three occurrences, CN, MS and AMR in summer 2010 are listed in S2 Table. Out of these nine loci, *Xpsmp716* showed maximum number of 43 significant associations (15 GCA associations and 28 SCA associations) with all traits except TN followed by *Xpsmp2076* showed 11 (3 were GCA associations and 8 were SCA associations) significant associations with 6 traits (FT, GY, DSY, GNP, PHI, and GHI). The current results revealed the phenomenon of pleiotropism, as well as the polygenic nature of combining ability as one locus was linked with many traits and also many loci, were linked with the same trait. For example, locus *Xpsmp716 was* linked to GCA values of FT, PN, DSY and VGI in CN and whereas GCA values of FT associated with both *Xpsmp716* and *Xpsmp2076* in MS.

The present study is the first of its kind to identify QTLs associated with combining ability (GCA and SCA values of traits) in pearl millet whereas earlier reports of QTL studies were based on performance per se of lines or their test crosses (absolute phenotypic values of traits). Hence our results could only be compared with the previous QTLs identified based on absolute phenotypic values. On LG3, *Xpsmp2214* and *Xpsmp2249* were linked with GCA values of GY with additive effects in MS environment in the current study. Bidinger et al. [2] identified QTLs on this LG3 for grain yield, grain mass, harvest index, and panicle harvest index at marker interval *Xpsmp108*, *Xpsmp2070* and *Xpsmp2214*. Yadav et al. [6] reported the harvest index and panicle harvest index QTL on LG3 associated with marker interval of *Xpsmp325*-*Xpsmp2070*. However, both of these studies reported that the ICMB 841 alleles at these QTLs on LG3 contributed favorably for these traits. In MS conditions, *Xpsmp716* on LG4 was significantly associated with GCA values of FT, PN, PY, DSY, BM and VGI and SCA values of PL, PD, and TGM. These results are an agreement with reports of Yadav et al. [5]. They reported *Xpsmp716* as QTL marker for panicle number and biomass yield under late drought stress environment in F_3_ mapping population derived from H 77/833-2 and PRLT 2/89-33. Yadav et al. [6] mapped QTL on LG7 related to a genomic region between markers *Xpsmp2074* and *Xpsmp2027* from 863B for grain yield, harvest index, and panicle harvest index. This QTL was environment-specific and contributed to grain yield only in the stress environment. They reported stover yield QTL on LG7 at the same genomic region but the favorable alleles were from ICMB 841. In the present study, *Xpsmp2074* was linked with GCA of panicle length but only in control treatments.

The grain yield QTLs for irrigated and moisture stress conditions in this study are linked to different morpho-physiological traits like panicle length (PL), panicle diameter (PD), 1000-grain mass (TGM), panicle harvest index (PHI), and grain harvest index (GHI). All these traits map together at two chromosome intervals with *Xpsmp2214* and *Xpsmp224*9 on LG3 for GCA (S2 Table, Fig 1), and grain number per panicle (GNP), panicle harvest index (PHI) and grain harvest index (GHI) also map together at two chromosome intervals with *Xpsmp716* and *Xpsmp2076* on LG4 for SCA (Fig 2). In addition, some of the phenological traits like flowering time (FT) mapped at *Xpsmp716* on LG4 for both GCA and SCA (Figs 1 and 2). These morpho-physiological traits have been known yield-contributing factors for grain yield and help in improving the dry matter partitioning to the grains and increase harvest index. On the other hand, flowering time is an important adaptation trait in different agro-ecologies for pearl millet. In the current study, the heritability of these traits (80–90%) was more than that of GY (73%). These traits often have higher heritability over grain yield allowing greater phenotypic selection efficiencies.

## Conclusions

The use of CSSLs for identification of stable QTLs linked to agronomically important traits in pearl millet was demonstrated for the first time in this study. The identified combining ability loci linked with GCA and SCA values of traits under irrigated, moisture stress and across these two conditions may facilitate enhanced grain and stover yield. Following validation studies in diverse environments and genetic backgrounds, the loci for the GCA and SCA identified for different moisture regimes and those across moisture regimes (AMR) may be useful for pearl millet heterosis and varietal breeding programs for well-endowed and drought-prone ecologies using marker-assisted selection (MAS).

## Materials and methods

### Plant materials

A set of 85 CSSLs and three elite testers were used in this study. The CSSLs were developed from advanced backcross populations derived from ICMB 841 and 863B. ICMB 841 is an agronomically elite pearl millet maintainer line in several hybrids, used as the parent and 863B is a landrace and tolerant to drought, was the donor parent [42]. The three testers are morphologically and genetically diverse elite restorers *viz*., H 77/833-2, PPMI 301 and RIB 3135–18. The tester, H 77/833-2, is the male parent of a number of heat tolerant, very early flowering, more tillering and high yielding pearl millet hybrids, including HHB 67 (843A × H 77/833-2) [53]) developed at Haryana Agricultural University, Hisar. HHB 67, which was extensively cultivated in Haryana and the Thar Desert margins of Rajasthan in north-western India. The tester, PPMI 301 is sensitive to terminal moisture stress conditions. It is the male parent of released full-season hybrid Pusa 301 (841A × PPMI 301) developed at the Indian Agricultural Research Institute, New Delhi. The tester, RIB 3135–18 is more sensitive to drought. It is the male parent of certified full-season hybrid RHB127 (ICMA 89111 × RIB 3135–18) developed at Rajasthan Agricultural University, Agricultural Research Station, Durgapura. During summer 2009, testcross hybrids were produced by dusting bulk pollen from each of the three testers on receptive stigmas (inside bagged panicles) of each of the CSSLs. Field trials of total 255 testcross hybrids from 85 CSSLs and three testers were conducted at ICRISAT-Patancheru during the 2010 summer season in two moisture regimes *i*.*e*., fully irrigated control conditions (CN) and early-onset moisture stress conditions (MS). During wet season 2010, field trial in fully irrigated control conditions (control) using only testcross hybrids of 85 CSSLs and H 77/833-2 was performed. As this season is rainy season perfect moisture stress conditions could not be maintained and field trials using other two testcross hybrids also could not be conducted due to insufficient seed material.

### Field trials

Test cross hybrid populations of 85 CSSLs of pearl millet were evaluated for 15 grain and stover yield-related traits for two seasons, summer and wet seasons 2010 under irrigated control (CN) and moisture stress (MS) conditions in field conditions. Field trials of testcross hybrids were conducted in three replications following alpha (incomplete block) designs to reduce replication variations in moisture stress treatments as much as possible. It was generally found that the effect of blocking was statistically significant, despite the general precautions taken in managing these experimental crops. Individual plots were one row of 4.0 m length with rows 0.6 m apart and net (harvested) plot area was one row of 3.0 m by 0.6 m (1.8 m^2^). Standard crop management procedures (described below) are followed to obtain uniform pre-flowering crop growth and start the moisture stress at a fixed crop developmental stage. Irrigation in the MS regime was terminated approximately one week before the flowering of the main shoot to initiate the stress around mid-flowering to affect both seed number and seed filling. The observations and measurements taken during the field trials were as follows,

- Flowering time (FT): Time of flowering was recorded as days from seedling emergence to stigma emergence in 75% of the main shoots in a plot.
- Plant height (PH): Plant height (cm) was measured from the base of the stem to the tip of the main culm panicle at the maturity. Data was recorded on three random plants from the middle of each row.
- Panicle length (PL): Length of the panicle (cm) was measured for the main culms of sample plants considered for plant height in each plot.
- Panicle diameter (PD): Panicle diameter (mm) was measured using Vernier calipers on all those panicles for which panicle length was recorded.
- Panicle number (PN): Panicles from the middle 3 m of one row of each plot were harvested and counted for all the entries.
- Effective tiller (TN): Number of productive tillers per plant was calculated by dividing PN by Plant count (Number of plants in the middle 3 m of one row of each plot was counted for all the entries)
- Panicle yield (PY): After harvesting was completed, panicles were put in an oven for 24 hours and dried at a temperature of 60°C. The dry weight of the panicles from each plot was then recorded before threshing.
- Grain yield (GY): Panicles were threshed and their grain cleaned. The weight of the grains from each plot was recorded.
- Dry stover yield (DSY): After panicles were harvested, the stems and the tillers were cut and put in an oven for 24 hours and dried at a temperature of 60°C and their dry weights were then recorded.
- 1000-grain mass (TGM): One hundred grains (g) were counted in two replicates and their weight was recorded for each entry and calculated for 1000 grain.
- Number of grains per panicle (GNP): Number of grains per panicle was derived from these primary data (= (100×GY)/ (PN×100 grain mass).
- Biomass yield (BM): Biomass yield was calculated for each plot as the sum of PY and DSY.
- Panicle harvest index (PHI): Panicle harvest index was calculated for each plot as the ratio of GY and PY.
- Grain harvest index (GHI): Grain harvest index was calculated for each plot as the ratio of GY and BM.
- Vegetative growth index (VGI): Vegetative growth index was calculated by using the formula, VGI = BM/ (FT+10).

The field trial conducted during summer 2010 was successful for both irrigated and drought treatments. However, during wet season 2010, field trial could be conducted in irrigated conditions only (due to the rainy season, drought conditions could not be maintained), using testcross hybrids of H 77/833-2.

### Genotyping

Genomic DNA was extracted from leaves of 85 CSSLs as well as their recurrent and donor parents using a high-throughput DNA extraction protocol described by Mace et al., [54]. Genotyping was done with a total of 88 SSR markers distributed across all seven linkage group (LG) of pearl millet. Among the SSR markers used, the *XPSMP and XPSMS* series genomic SSR and STS markers were developed by Qi et al. [55, 56] and Allouis et al. [57]. The *CTM* series genomic SSR markers were obtained from Budak et al. [58]. The *ICMP* (ICRISAT Millet Primer) series EST-SSR markers were developed by Senthilvel et al. [59]. The remaining *IPES* (ICRISAT Pearl millet EST Stress) markers were published by Rajaram et al. [60]. The forward primers were directly labeled with one of the four fluorescent dyes viz. Fam, Ned, Pet, and Vic along with reverse primers to facilitate high-throughput genotyping. PCR was performed in a 5 μl reaction volume containing 5 ng genomic DNA template, 0.2 p mole forward primer, 1 p mole of reverse primer, 0.5 μl of 2 mM dNTPs, 0.1 U Taq DNA polymerase and 0.5 μl of 10X PCR buffer in a Gen-Amp PCR system 9700 thermocycler (Applied Biosystems, USA). PCR conditions were as follows: denaturation at 94°C for 5 min, followed by 10 cycles of denaturation at 94°C for 15 s, annealing at 61°C to 51°C (touch-down cycles) for 30 s, and extension at 72°C for 30 s, followed by 40 cycles of denaturation at 94°C for 10 s, annealing at 54°C for 30 s, and extension at 72°C for 30 s, followed by final extension at 72°C for 20 min. PCR amplification was checked on 1.2% agarose gels and PCR products were separated by capillary electrophoresis on an ABI3730xl sequencer and their sizes were determined using GeneMapper v4.0 software (Applied Biosystems, USA).

### Statistical analysis

Line × tester analysis was performed using the software, GENSTAT 14th edition (2011). Analysis of variance (ANOVA), General combining ability (GCA) for each line and tester and specific combining ability (SCA) for each cross were estimated for each trait in CN, MS and across these two moisture regimes (AMR) as per Singh and Chaudhary [61].

The following formulae are used to compute SCA and GCA.

*GCA*(*Lines*)*gi* = (*Xi*..÷*tr*)−(*Xi*…÷*ltr*)

*GCA*(*Testers*)*gj* = *X.j.*÷*r*)−(*Xi*…÷*ltr*)

*SCA*(*Cross*)*Sij* = (*Xij*.÷*r*)−(*Xi*..÷*tr*)−(*X.j.*÷*lr*)+(*X*…÷*ltr*)

l = Line

t = Tester

r = Replication

Xi.. = Total of the ith line overall testers and replications

X… = Total of all hybrid combinations over all replications

X.j. = Total of jth tester overall lines and replications

Xij. = ijth combination total overall replications

The two moisture conditions *viz*., irrigated control (CN) and moisture stress (MS) conditions were treated as two random environments. QTL analyses for GCA and SCA across both environments were performed. General statistics for GCA and SCA values for all 15 traits and correlation among GCA values for all traits were calculated using Statistix 8.1 (Analytical Software Inc. USA). Correlation plots were prepared using R programming version 3.2.3 using package corrplot. Broad-sense heritability (H^2^, on entry mean basis) of all 15 grain and stover yield-related traits among testcross hybrids under CN and MS treatments was calculated using the software package GENSTAT (14th edition, 2011).

The details of linkage map construction were described by Kumari et al. [42] for 74 common markers out of a total of 88 markers. For the remaining 14 markers on LG1 from this study, map length was calculated using the best marker order determined by MAPMAKER/EXP (v3.0b) [62]. Map distances were estimated in Kosambi units. Single marker analysis (SMA) was done to find out the relationship between each marker and GCA or SCA values of each trait in CN, MS, and AMR using QTL cartographer. Only the loci with LOD >5.0 and the significance level of p <0.001 were considered as a significant locus. Linkage map showing the position of these significant marker loci was constructed using Map Chart version 2.2 software [63].

## Supporting information

S1 Table General combining ability (GCA) values for 15 grain and stover yield-related traits using testcross hybrids of 85 CSSLs and 3 testers under control (CN), and moisture stress (MS) and across two moisture regimes (AMR) in summer 2010. (XLS)

S2 Table The simultaneous effects of each combining ability loci on multiple traits in control (CN), and moisture stress (MS) and across two moisture regimes (AMR) in summer 2010. (XLS)

## Abbreviations

BM: Biomass yield (kg ha^-1^)
CSSLs: chromosome segment substitution lines
DArT: Diversity array technology
DFID: Department for International Development
DH: doubled haploid
DSY: Dry stover yield (kg ha^-1^)
FT: Time to 75% flowering (d)
GHI: Grain harvest index (%)
GNP: Grain number/panicle
GY: Grain yield (kg ha^-1^)
LG: linkage group
MAS: marker-assisted Selection
PD: Panicle diameter (cm)
PH: Plant height (cm)
PHI: Panicle harvest index (%)
PL: Panicle length (cm)
PN: Panicle number (000 ha^-1^)
PY: Panicle yield (kg ha^-1^)
RIL: recombinant inbred line
SSRs: simple sequence repeats
TGW: 1000-Grain mass (g)
TN: Tiller number per plant
VGI: Vegetative growth index (kg ha^-1^ d^-1^)
